# A “family-school-hospital” collaborative oral health management model for preschool children: a prospective single-arm pre-post intervention study

**DOI:** 10.3389/fdmed.2026.1926113

**Published:** 2026-10-05

**Authors:** Yi Zhang, Yiting Li, Yi Liu, Yuan Zhou, Wei Yin, Liwei Zheng

**Affiliations:** 1 State Key Laboratory of Oral Diseases, National Clinical Research Center for Oral Diseases, West China Hospital of Stomatology, Sichuan University, Chengdu, China; 2 Department of Stomatology, Chengdu Women’s and Children’s Central Hospital, Chengdu, China; 3 Department of Preventive Dentistry, West China Hospital of Stomatology, Sichuan University, Chengdu, China

**Keywords:** cooperative behavior, early childhood caries, health education, oral health, preschool

## Abstract

**Background:**

Early childhood caries (ECC) remains a global public health challenge, yet preschool children lack autonomy in health management and depend heavily on family and school environments. Population-based oral health management models have proven effective in other fields but are rarely implemented in Chinese preschool settings. This study aimed to establish and evaluate a “family-school-hospital” collaborative oral health management model for preschool children in Chengdu, China.

**Methods:**

The oral health management model for preschool children was established through population screening, baseline surveys, personalized oral health guidance, provision of diagnostic and treatment pathways, and the use of health behavior formation tools. The preliminary effects of promoting active health behavior formation were observed through oral health follow-up examinations and oral health-related questionnaire surveys conducted at 1-, 3-, and 6-month follow-ups.

**Results:**

After establishing the oral health management model for preschool children, a total of 421 children completed the six-month follow-up, with a loss to follow-up rate of 11.4%. The increase in the number of teeth restored, the decrease in plaque index, the increase in the number of children participating in fluoride application or pit and fissure sealing, the improvement in the number of children using fluoride toothpaste, the improvement in oral health-related quality of life scores, and the improvement in parental knowledge and attitude were all statistically significant.

**Conclusion:**

This collaborative model was associated with improved parental knowledge and attitudes, enhanced children's oral hygiene and restorative care, and better oral health-related quality of life. It offers a reference framework for preschool oral health management. Future randomized controlled trials with longer follow-up are warranted to confirm its preventive efficacy.

## 1 Introduction

Caries in primary teeth is the most common clinical condition encountered in pediatric dentistry and is highly prevalent worldwide (1–3). Unlike adults, young children lack the capacity for autonomous health management and rely heavily on parental supervision to safeguard their oral health. Parental oral health knowledge, attitudes, and practices directly influence children's oral hygiene habits and perceptions (4). Early childhood caries (ECC) is significantly correlated with family oral health behaviors. Kindergartens serve as the primary environment for preschool children's daily life and learning. Implementing oral health management programs within kindergartens has been shown to significantly improve children's toothbrushing skills, effectively reduce the incidence and mean number of decayed primary teeth, and increase the restoration rate (5, 6).

Population-based health management approaches have proven effective in controlling disease onset and progression, substantially reducing healthcare expenditures (7, 8). Caries prevention, specifically, can lower patient visit frequencies, decrease costs for families and the state, alleviate strain on healthcare facilities, and enhance clinician efficiency (9). Establishing a population-based caries management system is crucial for mitigating the oral disease burden in children.

In the evolving digital Caries Management By Risk Assessment (CAMBRA) landscape, recent mobile applications were employed in oral health promotion interventions (10–12). However, preschool populations differ from adolescents and adults, as they depend on caregivers and are unable to engage autonomously in smartphone-based self-management. While digital tools offer scalability, their direct applicability to young children remains limited.

Therefore, this study aims to establish a “family-school-hospital” collaborative oral health management model, and observe its impact, thereby providing a reference for preschool oral health care.

## 2 Materials and methods

This study used a prospective single-arm pre-post intervention design. The protocol was approved by the Medical Ethics Committee of West China Hospital of Stomatology, Sichuan University (Approval No. WCHSIRB-2022-289) and was registered with the Chinese Clinical Trial Registry (Registration No. ChiCTR2300078093). This study did not include a concurrent control group. All participants received the intervention. This design was chosen for practical and ethical reasons. First, the intervention combined oral health education with clinical preventive and restorative care. Withholding such care from a control group of preschool children would raise ethical concerns. Second, kindergarten-based recruitment made individual randomization logistically difficult. Third, this study aimed to establish the feasibility and preliminary effectiveness of the collaborative model before planning a larger randomized trial.

### 2.1 Study population

The target population consisted of children aged 3–5 years and their parents. Age was calculated based on the date of birth.

Inclusion criteria: (1) Children aged 3–5 years; (2) Signed informed consent from children and their guardians; (3) Normal temporomandibular joint and stomatognathic system function.

Exclusion criteria: (1) Inability to tolerate or cooperate with the required intraoral examination or dental procedures despite standard behavioral guidance; (2) Currently participating in another oral health-related intervention study; (3) Currently receiving or had received within 3 months medications that may affect oral health or study outcomes, such as immunosuppressants, antiepileptic drugs, or calcium channel blockers.

G*Power was used to calculate the sample size. The sample size was estimated for a paired t-test with a two-tailed *α* of 0.05% and 80% power. Assuming a moderate effect size of 0.5 and a standard deviation of change scores of 4.0 (13), a minimum of 32 completers was required. To compensate for an expected attrition rate of 30%, we needed to recruit at least 46 participants.

A total of 475 children were enrolled at baseline. Of these, 421 completed the 6-month follow-up (attrition rate: 11.4%), meeting the requirements for single-arm pre-post intervention study analysis.

Post-hoc power assessment confirmed that the longitudinal GEE analyses were adequately powered. With a conservative design effect of 2.5–3.1 (assuming *ρ* = 0.5–0.7 and m = 4 repeated measures), the effective sample size ranged from 136 to 168 completers, substantially below the actual *N* = 421. The minimum detectable effect size at 80% power (Cohen's d = 0.24) was far smaller than the observed effects for all primary endpoints, corresponding to >99% power. Thus, the sample size provided more than four times the required effective sample, ensuring robust statistical power for all longitudinal analyses.

### 2.2 Methods

We proposed a comprehensive oral health management model for preschool children. The study design and timeline are illustrated in Figure 1.

**Figure 1:**
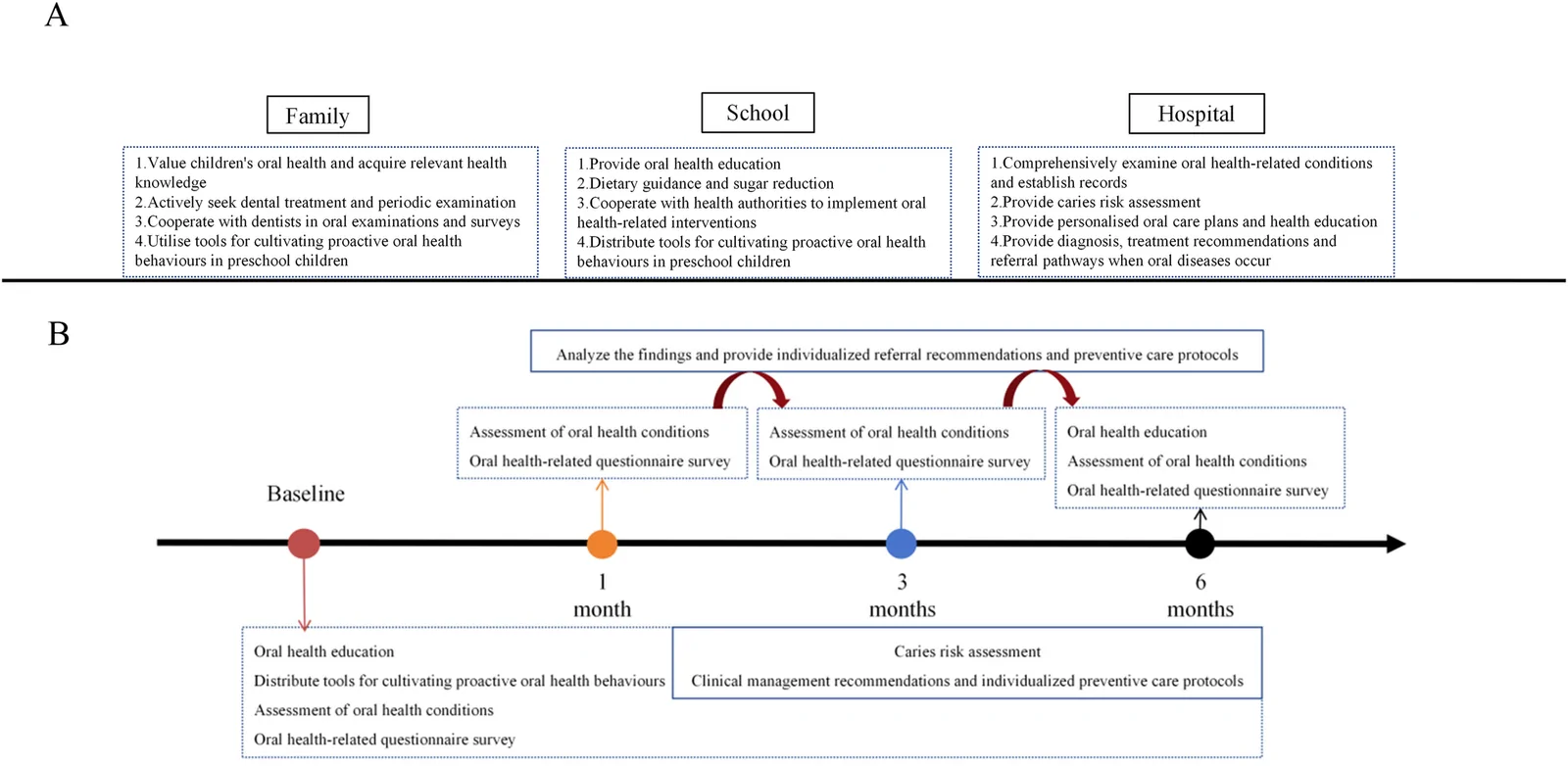
Flowchart. **(A)** “Family-School-Hospital” Oral Health Management Model; **(B)** Health Management Timeline.

The roles and responsibilities of families, schools, and healthcare institutions are illustrated in Figure 1A, while the specific activities at each time point are shown in Figure 1B. During the follow-up period, online educational push notifications, survey results, and personalized care plans were delivered to participants via the internet. After distributing the oral health behavior-formation tools, weekly online check-ins were conducted to monitor participants’ adherence to the recommended routines.

#### 2.2.1 Oral health status assessment

Caries-related indicators were assessed using criteria from the WHO *Oral Health Surveys: Basic Methods*. Decayed, missing and filled teeth (dmft) were recorded. Tooth demineralization and oral hygiene status were recorded to evaluate children's caries risk. Oral hygiene status was assessed using the Silness-Loe Plaque Index (PLI).

Each site was scored as follows:

0 = No plaque in the gingival area.
1 = A film of plaque adhering to the free gingival margin and adjacent area of the tooth, which may be recognized only by running a probe across the tooth surface.
2 = Moderate accumulation of soft deposits within the gingival pocket or on the tooth and gingival margin, which may be seen with the naked eye.
3 = Abundance of soft matter within the gingival pocket or on the tooth and gingival margin.

Caries prevalence rate: the percentage of participants with a dmft score greater than zero, calculated as (number of participants with dmft > 0/total participants) × 100%.

Restoration rate: the proportion of decayed, missing, or filled teeth that had been restored, calculated as [number of filled teeth (ft)/total dmft score] × 100%.

#### 2.2.2 Oral health-related questionnaire survey

The questionnaire covered children's demographic data, dietary habits, oral hygiene practices, parental oral health knowledge, and parental attitudes toward oral health. Furthermore, the Early Childhood Oral Health Impact Scale (ECOHIS) (14) was employed to assess the multidimensional impact of children's oral health on the child and their family. The total score ranges from 0 to 52. A higher score indicates a greater negative impact of oral health on the child's quality of life. For this study, we reported the data using Oral Health-Related Quality of Life (OHRQoL) scores (converted ECOHIS, 0–52, higher = better).

#### 2.2.3 Caries risk assessment

We modified the caries risk assessment tool for children from the American Academy of Pediatric Dentistry (AAPD) (15) to explore various risk and protective factors potentially affecting oral health. Caries-risk Assessment Form for 0–5 Years Old and Example of a Caries Management Pathways for 0–5 Years Old were modified. There is no fluoridated water policy locally. So content related to fluoridated drinking water has been excluded.

#### 2.2.4 Oral health education and personalized guidance

Oral health education consisted of chair-side oral hygiene instruction and structured health guidance sessions. Personalized guidance was provided during each examination, supplemented by online educational articles.

(1) Oral Health Guidance Sessions. Course content covered the etiology, impact, and prevention of dental caries, proper brushing and flossing techniques, and dietary recommendations. Teaching methods included lectures, video education, live demonstrations, and interactive Q and A sessions.
(2) Online educational push notifications. Promotional articles addressed topics including the impact of caries, toothbrushing methods, flossing, dental staining, fluoride application, pit and fissure sealants, emergency management of dental trauma, preformed crowns, and space maintainers.
(3) Personalized Guidance. Recommendations regarding dental visits, oral hygiene practices, fluoride application, and sealants were tailored based on caries risk assessment, knowledge/attitude questionnaire results, and clinical examination findings (Table 1). During examinations, parents were shown the specific teeth requiring treatment or enhanced cleaning. The dentist used a standardized set of visual aids: a dental model, and a printed brushing guide. Chair-side oral health instruction lasted about 5–10 min. Dentists manually write online feedback based on templates, using the results of children's oral examinations and parents’ questionnaire responses. Targeted feedback was provided online to highlight areas for improvement (e.g., reducing snack intake, using fluoride toothpaste).

**Table 1:** Oral health recommendations based on caries risk level.

Caries risk level	Fluoride varnish application	Dietary guidance	Pit and fissure sealants	Restorative management
Low	Not indicated	Not indicated	Recommended	Regular monitoring
Medium	Every 6 months	Indicated	Recommended	Monitor for early carious lesions
High	Every 3 months	Indicated	Recommended	Restore carious teeth

#### 2.2.5 Fostering proactive oral health behaviors

A tailored toolkit for preschool children (Figure 2) was developed to support family-based oral health management. It incorporated AAPD strategies for ECC prevention (16) and addressed behavioral guidance, dietary advice, health education, and habit formation, aiming to engage both parents and children in cultivating proactive oral health behaviors.

**Figure 2:**
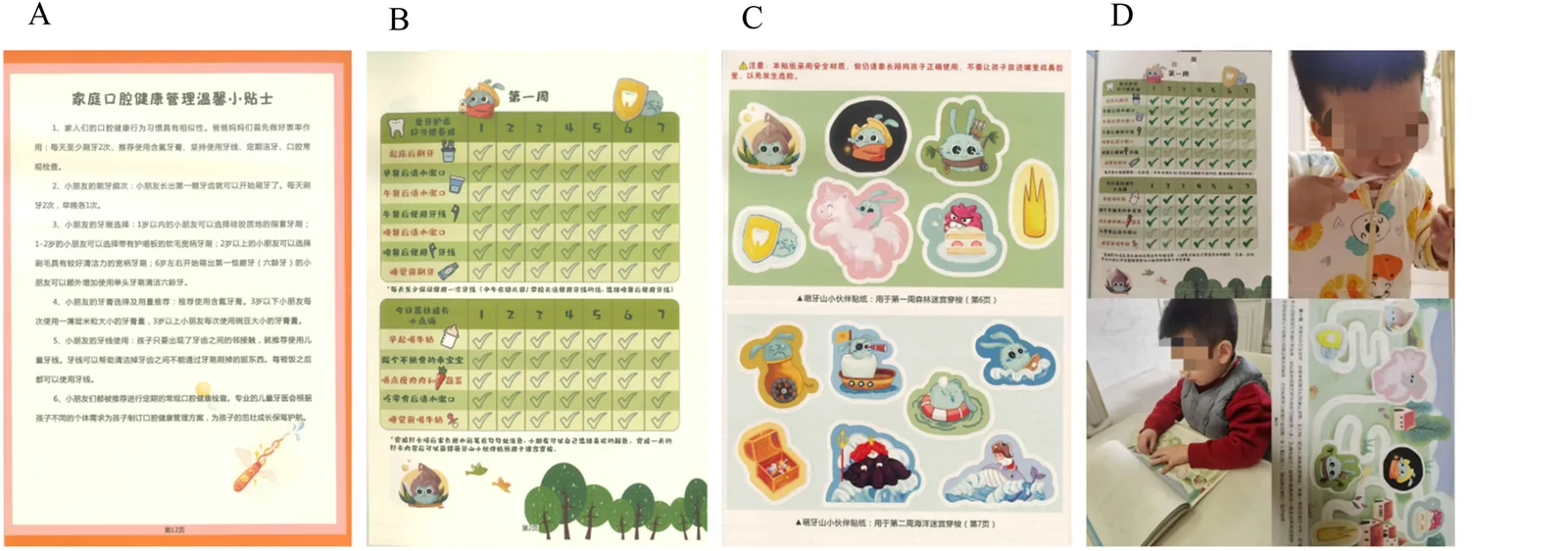
Toolkit for fostering proactive oral health behaviors in preschool children. **(A)** Family Oral Health Management Tips; 1. Family members’ oral health habits tend to be similar. Parents should lead by example: brush teeth at least twice daily, use fluoride toothpaste, floss regularly, get professional cleanings, and have routine dental check-ups. 2. Brushing frequency for children: Children can start brushing as soon as their first tooth erupts. Brush twice a day, once in the morning and once in the evening. 3. Toothbrush selection for children: Children under 1 year old can use silicone finger brushes; children aged 1−2 years can use soft-bristled wide-handled toothbrushes with a tongue guard; children over 2 years old can use wide-handled toothbrushes with good cleaning bristles; children around 6 years old who start erupting their first permanent molars (six-year molars) can additionally use single-tuft brushes to clean these molars. 4. Toothpaste selection and dosage recommendations for children: Fluoride toothpaste is recommended. Children under 3 years old should use a thin smear (rice-grain size) each time; children over 3 years old should use a pea-sized amount each time. 5. Flossing for children: Children's dental floss is recommended as soon as adjacent teeth make contact. Floss helps clean between teeth where a toothbrush cannot reach. It can be used after every meal. 6. Children are recommended to have regular routine oral health examinations. Professional pediatric dentists will develop personalized oral health management plans based on each child's individual needs, safeguarding their healthy growth and development. **(B)** Children's Behavioral and Dietary Guidance Charts; Oral Health Habits Checklist (Brush teeth after waking up; Rinse with water after breakfast; Rinse with water after lunch; Use dental floss after lunch; Rinse with water after dinner; Use dental floss after dinner; Brush teeth before bedtime). Use dental floss at least once a day. (If flossing is not possible at noon in kindergarten/school, floss after dinner instead.). **(C)** Positive Reinforcement Reward Stickers; **(D)** Online Check-in for Habit Tracking.

The main components of the toolkit included:

1. Family Oral Health Management Tips (Figure 2A): Emphasized the parental role as role models and supervisors, the importance of regular dental check-ups, recommendations for fluoride toothpaste use, brushing at least twice daily, flossing, age-appropriate toothbrush selection, and correct toothpaste amounts.
2. Children's Behavioral and Dietary Guidance Charts (Figure 2B): Encouraged habits like morning and evening brushing, post-meal rinsing, and flossing. Dietary guidance included balanced nutrition, exercising chewing function, and rinsing after snacks.
3. Positive Reinforcement Tools (Figure 2C): Designed based on preschool children's psychological characteristics to attract their engagement. Positive reinforcement encouraged children's performance, fostering good oral health attitudes and behaviors.

Before the intervention began, all participating kindergarten teachers attended a training session conducted by the research team. The training covered: (a) the purpose of the study; (b) how to use the toolkit and explain it to parents; (c) how to monitor weekly WeChat check-ins; (d) when and how to send reminders.

Adherence was monitored via online check-ins (Figure 2D), while epidemiological oral examinations assessed the outcomes of proactive health behaviors. Parents submitted photos of the completed weekly tracking chart (Figure 2B) to a dedicated WeChat group managed by the kindergarten teacher. At the 1-month clinical follow-up visit, parents brought the paper toolkit to the clinic. At each follow-up, children with excellent compliance were recognized and rewarded, reinforcing their motivation and serving as role models for their peers.

#### 2.2.6 Statistical analysis

Children's oral health data and parental questionnaire responses were double-entered into Excel software. Statistical analyses were performed using Statistical Product and Service Solutions (SPSS version 26.0). As caries-related data and questionnaire scores typically did not follow a normal distribution, the Mann–Whitney U test was used for between-group comparisons, and the Kruskal–Wallis H test for multi-group comparisons. Chi-square tests were applied to compare categorical variables such as caries prevalence, brushing frequency, and flossing rate. We applied generalized estimating equations (GEE) to compare differences in oral clinical parameters and questionnaire outcomes across baseline and follow-ups. In the model specification, decayed teeth (dt), filled teeth (ft), and dmft were fitted with a negative binomial log-link distribution, while OHRQoL, Knowledge and attitude scores and PLI were fitted with a Gamma log-link distribution. Additionally, a binomial logit GEE was adopted for oral health related habits, such as tooth-brushing frequency and flossing.

#### 2.2.7 Examiner calibration and reliability

Clinical examinations were performed by three trained pediatric dentists (Z.Y., L.Y.T., and L.Y.).

During the research, 20 children were randomly selected for re-examination. Examiners were blinded to each other's recordings. Inter-examiner reliability was assessed using Cohen's Kappa for dt and ft and weighted Kappa (linear weighting) for the PLI. Pairwise Kappa values were calculated for all three examiner pairs. Inter-examiner agreement was almost perfect. The mean Cohen's Kappa for dt was 0.851, while 0.847 for ft. For PLI, the mean weighted Kappa was 0.850.

## 3 Results

### 3.1 Baseline characteristics

A total of 475 children were enrolled. Valid questionnaires were recovered from 400 parents (response rate: 84.2%), which is moderate compared to similar studies (17, 18). Notably, the proportion of severe early childhood caries (SECC) children completing the questionnaire (63.2%) was lower than that of caries-free (90.3%) or ECC (91.6%) children.

As shown in Table 2, girls had significantly lower PLI scores than boys. No statistically significant differences were found between sexes regarding caries prevalence, dt, mt, ft, or dmft. Similarly, no sex-based differences were observed in OHRQoL scores, knowledge/attitude scores, dietary habits (snack frequency, nighttime bottle after brushing), or preventive/cleaning practices (regular fluoride/sealant use, fluoride toothpaste use, brushing frequency, and flossing) (Table 3).

**Table 2:** Caries status by sex.

Indicators	Male	Female	*p*-value
Proportion of participants (%)	49.3	50.7	
Caries prevalence rate (%)	57.3	56.4	0.855
Decayed teeth (dt)	3.20 ± 4.10	2.57 ± 3.86	0.127
Missing teeth (mt)	0.00 ± 0.00	0.03 ± 0.41	0.326
Filled teeth (ft)	0.34 ± 1.34	0.23 ± 0.86	0.741
dmft index	3.50 ± 4.42	2.78 ± 3.99	0.139
Plaque index (PLI)^a^	1.41 ± 0.67	1.28 ± 0.70	0.037

a Statistically significant difference between groups (*p* < 0.05).

**Table 3:** Parental questionnaire results by child's sex.

Indicators		Male	Female	*p*-value
Proportion of participants (%)		49.5	50.5	
Oral health-related quality of life (OHRQoL)		47.52 ± 5.39	47.51 ± 5.18	0.95
Knowledge and attitudes		11.26 ± 2.18	11.25 ± 2.09	0.84
Snack frequency (%)	0 times	14.1	8.9	0.661
	1–2 times	69.7	72.8	
	3 times	16.2	18.3	
Nighttime bottle (%)	Yes	24.7	26.7	0.734
	No	75.3	73.3	
Regular fluoride varnish/pit and fissure sealants (%)	Yes	47	51	0.481
	No	53	49	
Fluoride toothpaste use (%)	Yes	62.6	68.3	0.275
	No	37.4	31.7	
Toothbrushing frequency (%)	Once	31.8	31.2	0.978
	Twice or more	68.2	68.8	
Floss use (%)	Yes	29.8	33.2	0.537
	No	70.2	66.8	

The 5-year-old group exhibited significantly higher caries prevalence, dt, ft, dmft, and PLI compared to both the 3- and 4-year-old groups (Table 4). No significant differences were found between the 3- and 4-year-old groups. This may be partly explained by the higher proportion of SECC children in the 5-year-old group (33%) compared to the 3-year-old (19.4%) and 4-year-old (14%) groups. Older children were also more likely to have received regular fluoride applications/sealants, used fluoride toothpaste, and brushed twice or more daily (Table 5).

**Table 4:** Caries status by age.

Indicators	3-year-old	4-year-old	5-year-old	*p*-value
Proportion of participants (%)	19.2	34.1	46.7	
Caries prevalence rate (%)*	41.8	50.6	67.6	3y vs. 4y *p* = 0.175 3y vs. 5y *p* < 0.001 4y vs. 5y *p* = 0.001
dt*	2.05 ± 3.48	1.96 ± 2.96	3.85 ± 4.55	3y vs. 4y *p* = 0.404 3y vs. 5y *p* < 0.001 4y vs. 5y *p* < 0.001
mt	0.00 ± 0.00	0.00 ± 0.00	0.03 ± 0.42	
ft*	0.06 ± 0.40	0.09 ± 0.68	0.51 ± 1.47	3y vs. 4y *p* = 0.878 3y vs. 5y *p* = 0.003 4y vs. 5y *p* < 0.001
dmft*	2.11 ± 3.46	2.04 ± 3.01	4.29 ± 4.87	3y vs. 4y *p* = 0.499 3y vs. 5y *p* < 0.001 4y vs. 5y *p* < 0.001
PLI*	1.17 ± 0.80	1.19 ± 0.60	1.54 ± 0.64	3y vs. 4y *p* = 0.858 3y vs. 5y *p* < 0.001 4y vs. 5y *p* < 0.001

* Statistically significant differences (*p* < 0.05).

**Table 5:** Parental questionnaire results by child's age.

Indicators		3-year-old	4-year-old	5-year-old	*p*-value
Proportion of participants (%)		18	35	47	
OHRQoL		47.56 ± 5.65	48.27 ± 4.45	46.83 ± 5.63	0.16
Knowledge and attitudes		11.09 ± 1.95	11.45 ± 1.99	11.17 ± 2.38	0.34
Snack frequency (%)	0 times	6.9	7.1	15.4	0.564
	1–2 times	75	73.6	69.2	
	3 times	18.1	19.3	15.4	
Nighttime bottle (%)	Yes	34.7	25	22.9	0.143
	No	65.3	75	77.1	
Regular fluoride varnish/pit and fissure sealants (%)*	Yes	34.7	50	53.7	3y vs.4y *p* = 0.049 3y vs.5y *p* = 0.009 4y vs.5y *p* = 0.578
	No	65.3	50	46.3	
Fluoride toothpaste use (%)*	Yes	55.6	58.6	77.7	3y vs.4y *p* = 0.784 3y vs.5y *p* < 0.001 4y vs.5y *p* < 0.001
	No	44.4	41.4	22.3	
Toothbrushing frequency (%)*	Once	52.8	27.1	26.1	3y vs.4y *p* < 0.001 3y vs.5y *p* < 0.001 4y vs.5y *p* = 0.92
	Twice or more	47.2	72.9	73.9	
Floss use (%)	Yes	29.2	26.4	36.2	0.153
	No	70.8	73.6	63.8	

* Statistically significant differences (*p* < 0.05).

Analysis of parental questionnaires based on caries severity revealed that children with SECC had significantly lower OHRQoL scores, lower parental knowledge/attitude scores, lower rates of fluoride toothpaste use, lower rates of brushing twice or more daily, and higher rates of consuming snacks three times or more daily (Table 6).

**Table 6:** Parental questionnaire results by caries severity.

Indicators		Caries free (NC)	Early childhood caries (ECC)	Severe early childhood caries (SECC)	*p*-value
Proportion of participants (%)		46.5	35.5	18	
OHRQoL*		49.49 ± 3.84	46.87 ± 4.30	41.59 ± 6.42	NC vs. ECC *p* < 0.01 NC vs. SECC *p* < 0.01 ECC vs. SECC *p* < 0.01
Knowledge and attitudes*		11.32 ± 1.96	11.37 ± 2.18	10.59 ± 2.17	NC vs. ECC *p* > 0.99 NC vs. SECC *p* < 0.01 ECC vs. SECC *p* = 0.04
Snack frequency (%)*	0 times	11.8	12.7	8.3	NC vs. ECC *p* = 0.795 NC vs. SECC *p* = 0.002 ECC vs. SECC *p* = 0.009
	1–2 times	73.7	71.8	63.9	
	3 times	14.5	15.5	27.8	
Nighttime bottle (%)	Yes	27.4	19.7	33.3	0.077
	No	72.6	80.3	66.7	
Regular fluoride varnish/pit and fissure sealants (%)	Yes	51.1	49.3	40.3	0.29
	No	48.9	50.7	59.7	
Fluoride toothpaste use (%)*	Yes	68.8	70.4	47.2	NC vs. ECC *p* = 0.848 NC vs. SECC *p* = 0.002 ECC vs. SECC *p* = 0.002
	No	31.2	29.6	52.8	
Toothbrushing frequency (%)*	Once	32.8	21.1	47.2	NC vs. ECC *p* = 0.027 NC vs. SECC *p* = 0.044 ECC vs. SECC *p* < 0.001
	Twice or more	67.2	78.9	52.8	
Floss use (%)	Yes	27.4	33.8	37.5	0.225
	No	72.6	66.2	62.5	

* Statistically significant differences (*p* < 0.05).

### 3.2 Follow-up outcomes

Of the initial cohort, 452 children completed the 1-month follow-up, 427 completed the 3-month follow-up, and 421 completed the 6-month follow-up. The 54 children lost to follow-up did not differ significantly from completers in age, sex, baseline caries status, dmft, PLI, or OHRQoL scores. The overall attrition rate was 11.4%, consistent with similar studies. The main reasons for loss to follow-up included children's illness, family relocation, and parental time constraints. At the 6-month follow-up, 366 questionnaires were returned, with a loss-to-follow-up rate of 8.5%. Non-responders did not differ significantly from responders in any baseline characteristic.

#### 3.2.1 Changes in clinical oral health parameters post-intervention

After six months of management, the mean dt showed a continuous decreasing trend. The restorative care index increased substantially from 8.33% at baseline to 34.29% at six months, indicating significant restorative treatment activity during follow-up. PLI significantly decreased from 1.35 at baseline to 0.87 at one month and remained low at subsequent follow-ups, suggesting marked improvement in oral hygiene. However, caries prevalence and dmft slightly increased at the 3- and 6-month follow-ups compared to baseline (Table 7, Figure 3).

**Table 7:** Changes in oral health parameters over time.

Indicators	Baseline	1 month	3 months	6 months
Caries prevalence rate (%)*	56.90	58.33	62.38	60.95
Restoration rate (%)*	8.33	19.52	30.48	34.29
dt*	2.87 ± 3.99	2.55 ± 3.88	2.34 ± 3.66	2.21 ± 3.44
mt	0.00 ± 0.00	0.00 ± 0.00	0.02 ± 0.23	0.03 ± 0.26
ft*	0.28 ± 1.12	0.56 ± 1.38	1.00 ± 1.89	1.15 ± 2.00
dmft*	3.13 ± 4.22	3.05 ± 4.14	3.28 ± 4.16	3.37 ± 4.22
PLI*	1.35 ± 0.68	0.87 ± 0.67	0.66 ± 0.78	0.72 ± 0.73

* Statistically significant differences (*p* < 0.05).

**Figure 3:**
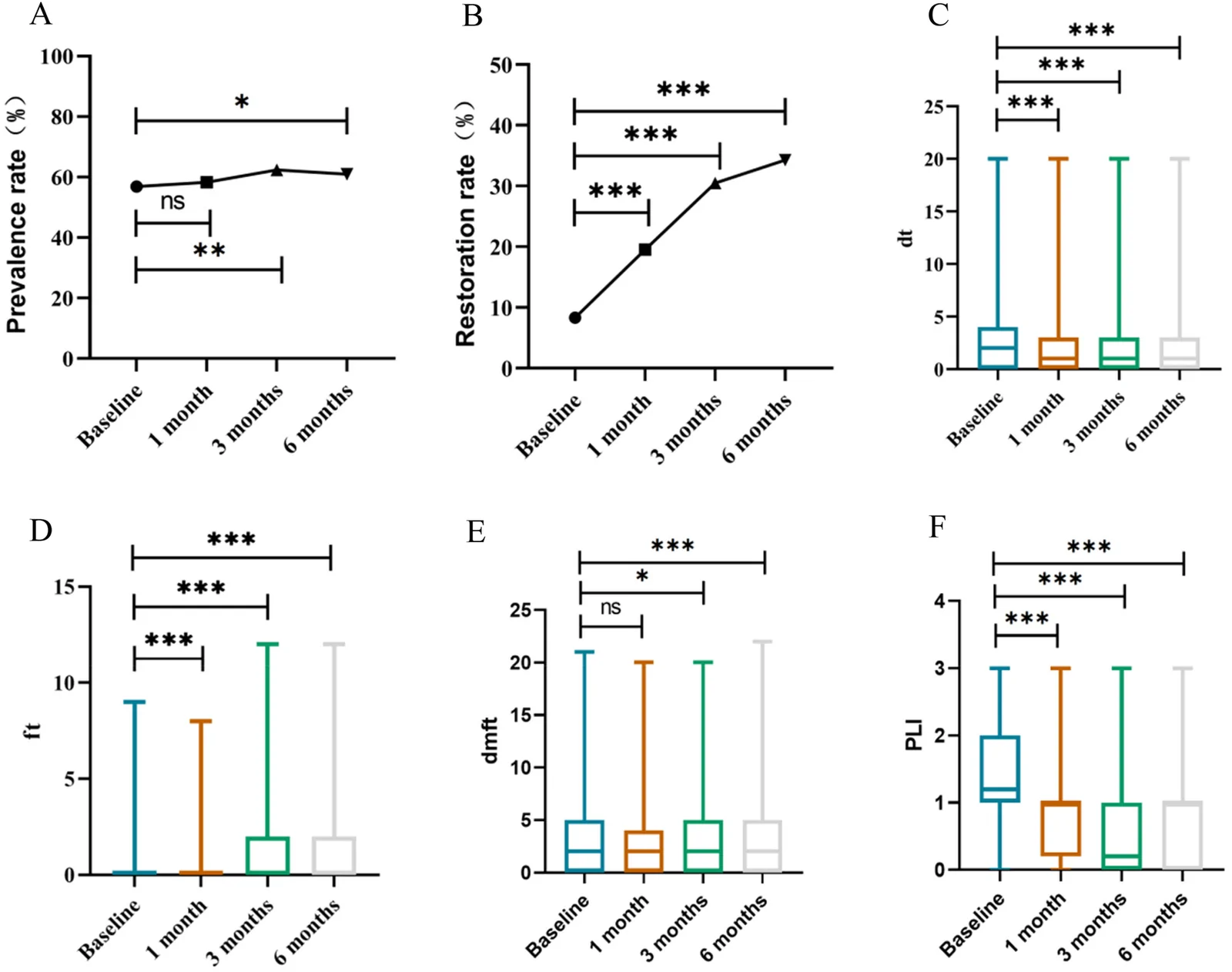
Changes in oral health parameters over time. **(A)** Comparison of prevalence rate vs. baseline; **(B)** Comparison of restoration rate vs. baseline; **(C)** Comparison of decayed teeth (dt) vs. baseline; **(D)** Comparison of filled teeth (ft) vs. baseline; **(E)** Comparison of dmft vs. baseline; **(F)** Comparison of plaque index (PLI) vs. baseline.

#### 3.2.2 Changes in questionnaire outcomes post-intervention

As shown in Table 8 and Figure 4, children's OHRQoL scores significantly improved following the intervention. Parental knowledge/attitude scores also increased significantly. The proportion of children reported to have received recent fluoride applications or sealants rose substantially at 1 month, remained elevated at 3 months, but showed a slight decline at 6 months. Fluoride toothpaste use increased markedly at 1 month and remained at a high level. Notably, flossing rates increased significantly at 1 month but returned to baseline levels at 3 months. This might reflect low compliance in younger children and/or a lack of parental persistence. After reinforcing the importance of flossing and providing additional instruction at the 3-month visit, the proportion of children using floss increased significantly at the 6-month follow-up. No statistically significant changes were observed in snack frequency, nighttime bottle, or brushing frequency.

**Table 8:** Changes in questionnaire outcomes over time.

Indicators		Baseline	1 month	3 months	6 months
Number of participants		400	389	371	366
OHRQoL*		47.51 ± 5.27	48.68 ± 4.73	49.13 ± 4.93	49.95 ± 3.54
Knowledge and attitudes*		11.26 ± 2.13	11.85 ± 1.60	12.21 ± 1.26	12.03 ± 1.54
Snack frequency (%)	0 times	11.5	13.3	10.5	13.1
	1–2 times	71.3	67.9	72.5	68
	3 times	17.2	18.8	17	18.9
Nighttime bottle (%)	Yes	25.8	21.9	19.4	20.2
	No	74.2	78.1	80.6	79.8
Regular fluoride varnish/pit and fissure sealants (%)*	Yes	49	81.7	82.2	55.7
	No	51	18.3	17.8	44.3
Fluoride toothpaste use (%)*	Yes	65.5	81.7	82.2	83.3
	No	34.5	18.3	17.8	16.7
Toothbrushing frequency (%)	Once	31.2	30.1	27.8	29.2
	Twice or more	68.8	69.9	72.2	70.8
Floss use (%)*	Yes	31.5	45.8	32.6	41.5
	No	68.5	54.2	67.4	58.5

* Statistically significant differences (*p* < 0.05).

**Figure 4:**
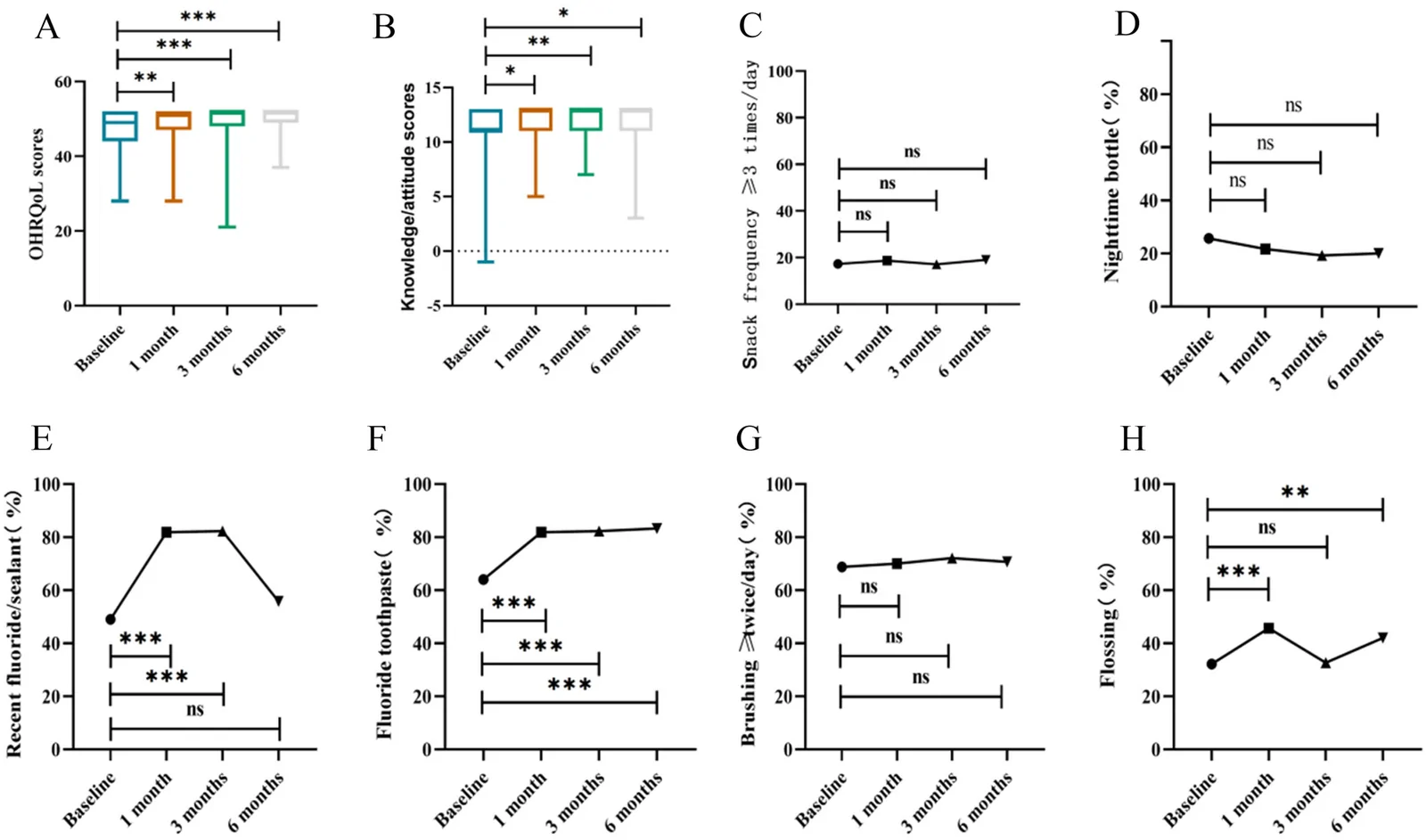
Changes in questionnaire outcomes over time. **(A)** OHRQoL scores vs. baseline; **(B)** Knowledge/Attitude scores vs. baseline; **(C)** Proportion of children with snack frequency ≥3 times/day vs. baseline; **(D)** Proportion with nighttime bottle vs. baseline; **(E)** Proportion with recent fluoride/sealant use vs. baseline; **(F)** Proportion using fluoride toothpaste vs. baseline; **(G)** Proportion brushing ≥ twice/day vs. baseline; **(H)** Proportion flossing vs. baseline.

#### 3.2.3 Changes in parental knowledge and attitudes

At baseline, all parents agreed that oral health is important and acknowledged the importance of protecting the “first permanent molars” and the necessity of regular dental check-ups. Only one parent disagreed with the statement that “parents are the primary responsible person for their child's oral health.” Only 1.9% of parents believed that “decayed primary teeth do not need treatment.” A large majority (91%) were aware of the preventive effect of fluorides. Following the intervention, awareness of pit and fissure sealing sealants improved by nearly 10%, reaching a correct response rate of 85.5%. (Figure 5, Supplementary Table).

**Figure 5:**
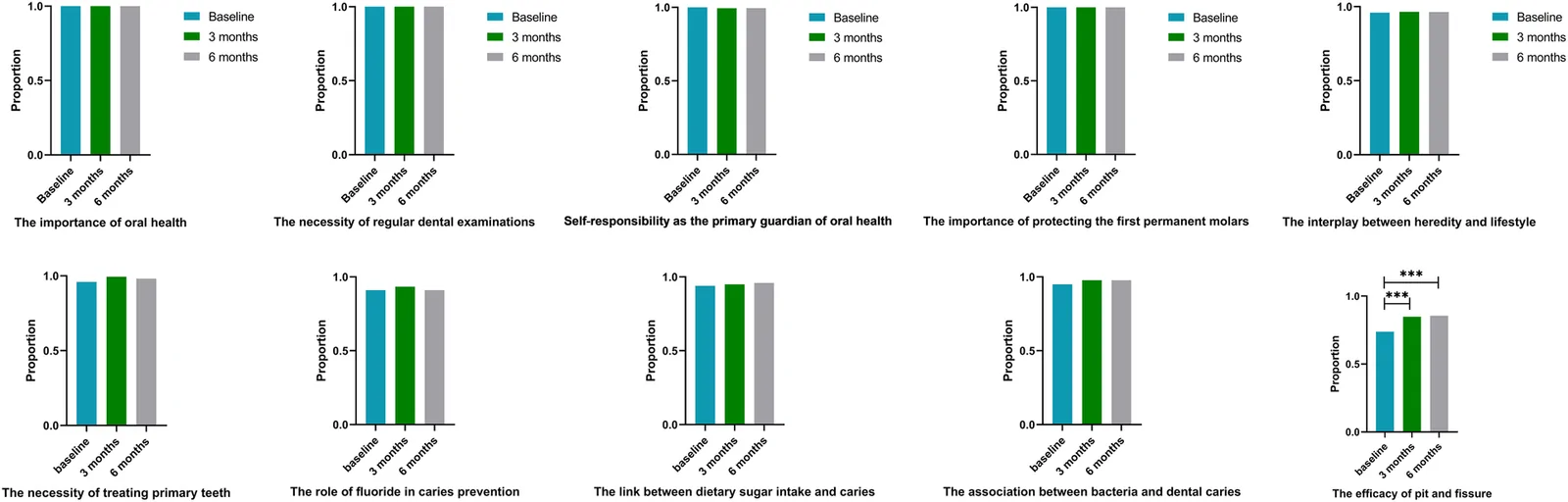
Changes in parental knowledge and attitudes.

#### 3.2.4 Changes in OHRQoL (ECOHIS subscales)

Many adverse effects were observed to significantly decrease, such as financial burden, sleep disruption, school absenteeism, emotional distress. (Figure 6, Supplementary Table).

**Figure 6:**
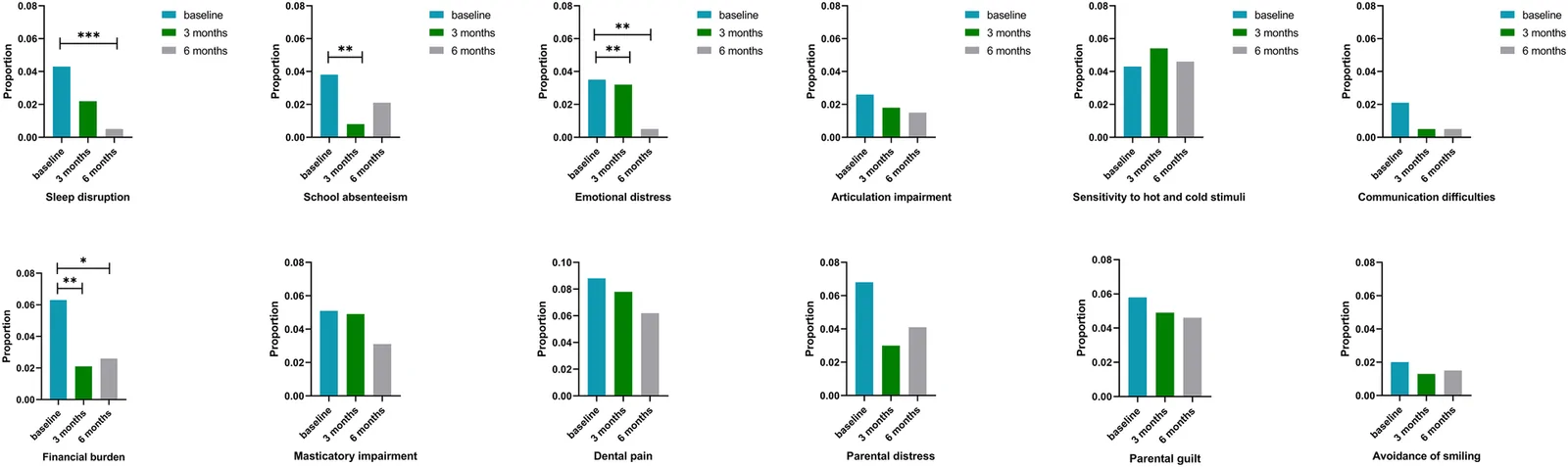
Changes in OHRQoL.

## 4 Discussion

This study successfully established a “family-school-hospital” collaborative oral health management model for preschool children. Over six months, this model was associated with increases in parental attention to preschool oral health, their oral health knowledge and attitudes. In addition, it may also have contributed to proactive oral health behaviors in children, and the enhancements in the children's oral health-related quality of life.

Data from the 4th National Oral Health Survey of China indicated caries prevalence rates of 50.8% in 3-year-olds, 63.6% in 4-year-olds, and 71.9% in 5-year-olds (19). These figures, showing that nearly half of 3-year-old children are already affected, underscore the need to shift the focus of caries prevention earlier and intervene in preschool children's oral health proactively. The American Academy of Pediatrics introduced the Medical Home concept, and subsequent research demonstrated that population-based management systems effectively control disease progression and reduce healthcare expenditures (20–22). However, similar population health management systems are not yet widely implemented in China. Our study addresses this gap by establishing a localized collaborative model that integrates family, school, and healthcare resources.

Children inherently lack the capacity for self-directed health management. Parental oral health beliefs and practices profoundly influence children's habits (4). Children's brushing and flossing habits, as well as their perceptions of oral health, are largely shaped by their family environment. Furthermore, ECC is closely linked to family oral hygiene behaviors. Studies have shown that children from mothers with low educational levels and low-income families had a higher risk of developing SECC (23, 24). In our study, parents of SECC children had lower questionnaire return rates. While the SECC proportion was similar among those lost to follow-up, SECC children received less restorative care and showed smaller OHRQoL improvements. This may reflect that more educated adults are better able to recognize and obtain needed dental treatment (25). Alternatively, families with greater caries burden might underreport quality-of-life impacts at equivalent disease levels (26).

Kindergartens represent a key setting for preschool children's social and learning activities, making teachers influential figures. Effective kindergarten-based oral health programs can enhance children's brushing skills, reduce primary caries incidence and mean dmft, and increase restorative care ratios (27). Therefore, kindergartens play a crucial role in the oral health management model.

The family-centered oral health management model adopted in this study was associated with the enhancement of parental knowledge and attitudes toward children's oral health. Through a combination of on-site lectures, personalized chair-side guidance during examinations, and online educational content covering topics like caries etiology, prevention, brushing, flossing, and diet, we improved parental knowledge. After six months, parental awareness of pit and fissure sealing sealants increased from 73.8% to 85.5%. Studies have confirmed the efficacy of fissure sealants in reducing caries incidence (28, 29). Thus, by increasing parental awareness and uptake of sealants, we can potentially decrease caries prevalence in preschool children. Interestingly, despite 91% of parents acknowledging the caries-preventive effect of fluoride at baseline, only 65.5% of children used fluoride toothpaste. The American Dental Association recommends brushing with fluoride toothpaste once teeth erupt (30). Our explanations regarding the mechanism and safety of fluoride may have partly alleviated parental concerns. Consequently, the proportion of children using fluoride toothpaste increased significantly at follow-up. This demonstrates that improved parental knowledge and attitudes translated into the adoption of proactive oral health behaviors in their children.

In the context of the rapidly evolving digital landscape for caries management, it is pertinent to situate the present model within the broader spectrum of technology-based CAMBRA interventions. Recent developments include mobile applications such as the CAMBRA-students (31) and ToothSense (32) for adolescent populations and parent-oriented ECC prevention tools that employ push notifications and brushing reminders (10, 12). They demonstrate that technology-based CAMBRA interventions can be effective in pediatric populations, supporting the value of systematic, risk-based caries management. The target population of our study—preschool children, who are dependent on adult caregivers. They have limited capacity to engage autonomously with smartphone-delivered self-management tools. While CAMBRA-kids (33) was designed for the 0–5 age group, it remains primarily a parent-operated digital tool rather than a multi-stakeholder collaborative framework. These app-based approaches have not systematically involved kindergartens as partners in oral health management. Yet kindergartens are the primary setting where preschool children spend most of their daily life and learning. Our model addresses this gap by establishing a tripartite collaboration that actively involves families, schools, and healthcare institutions, using low-technology behavioral supports that are accessible to all families regardless of digital literacy. This finding implies that low-technology behavioral supports, when coupled with institutional partnerships, may constitute a cost-effective and culturally adaptable alternative to high-technology CAMBRA implementations, particularly in settings with variable resource availability. Future investigations might explore whether integrating this multi-component framework into a dedicated mobile application would yield additional gains in adherence and treatment outcomes.

This study may have contributed to proactive oral health behaviors among preschool children. Participation in our program led to a substantial increase in the rate of restorative treatment. The restorative care index rose from 8.33% at baseline to 34.29% at six months, and ft scores increased significantly. Preschool children are particularly susceptible to proximal caries in the anterior maxillary region. Shallow lesions in these areas may be managed non-restoratively with fluoride application and regular monitoring (34). At the one- and three-month follow-ups, we observed significant increases in the proportion of children who had received recent fluoride or sealant applications. Moreover, oral hygiene, as measured by PLI, improved markedly. Although brushing frequency did not change, the reduction in PLI suggests more effective plaque removal, likely resulting from improved cleaning techniques.

Furthermore, flossing rates increased at one month, indicating that the behavioral toolkit successfully promoted initial adoption. However, the subsequent decline at three months may be attributable to poor compliance in young children and/or a lack of parental consistency. After reinforcing the importance and method of flossing at the three-month visit, the flossing rate rose again at the six-month follow-up. This fluctuation pattern suggests that behavior maintenance requires repeated reinforcement (35) rather than one-time intervention, which has important implications for the design of future health management protocols.

It is interesting to acknowledge that despite improvements in oral hygiene and parental behaviors, caries prevalence and dmft slightly increased at the 3- and 6-month follow-ups compared to baseline. This phenomenon reflects the natural biology of dental caries. Several factors may explain this observation. First, the six-month follow-up period may be insufficient to observe the full preventive effect of the intervention. The natural progression of caries in preschool children can be rapid, particularly for proximal lesions in the maxillary incisors and occlusal surfaces of the mandibular molars (36). The International Caries Detection and Assessment System (ICDAS) provides the ability for dentists to visually detect and assess early lesions and provide longitudinal follow-up (37). An ICDAS-based study has shown that cavitation of lesions initially scored as 1 occurred across the entire four-year follow-up, whereas cavitation of lesions initially scored as 3 and 4 was concentrated within the first year (38). Second, the prevention of dental caries requires long-term practice. A systematic review showed that nonrestorative, noninvasive and microinvasive caries treatments can arrest or reverse existing noncavitated carious lesions (39). In our study, the proportion of children receiving regular fluoride applications or sealants declined from 82.2% at 3 months to 55.7% at 6 months. According to Table 1, only high-risk children need fluoride varnish every 3 months. By 6 months, many children who received fluoride at 1 or 3 months were not yet due for their next application. Pit and fissure sealants are typically applied once per tooth. Once sealed, teeth do not need re-sealing at 6 months. Preventive behaviors must be maintained continuously (40) rather than delivered as a one-time burst. This observation actually strengthens the argument that behavioral interventions require sustained long-term implementation before measurable reductions in caries incidence.

The ECOHIS, developed by Pahel et al. for 3–6 year-olds, assesses the impact of oral health on children and their families (14). It has been validated across several languages and demonstrates good reliability, validity, and utility (41, 42). Pahel et al. reported that higher ECOHIS scores indicated greater negative impact. In our study, we reverse-coded scores so that higher values represented better OHRQoL, consistent with the direction of improvement observed. Following six months of management, participants’ mean OHRQoL scores improved significantly. The effects on OHRQoL of preschool children are manifested in multiple domains, among which pain is the most salient. A Hong Kong study reported that a majority (75%) of 5-year-old children had never visited a dentist, and those who did often sought care for relief of pain (43). Regular dental check-ups facilitate early intervention before significant pain or pathology develops. Providing oral health education alone, without addressing clinical preventive and therapeutic needs, may be insufficient to reduce the caries burden effectively. Therefore, an integrated approach combining prevention and treatment is essential to improve OHRQoL in preschool children. Currently, most dental clinics and hospitals in China primarily serve adults, with a relative shortage of pediatric dental specialists. Establishing dedicated pediatric dental clinics can help alleviate children's anxiety and fear during treatment (44), ensuring more timely and effective management of their oral health needs.

This study has several limitations that should be considered when interpreting the findings. Above all, time effects, maturation effects and the Hawthorne effect cannot be ruled out due to the absence of a control group. First, the Hawthorne effect may have played a role. Parents and children knew they were in a study. Parents may have reported better behaviors than they actually practiced. Children may have brushed better during check-ins than on normal days. Second, maturation effects are likely. Dental caries in preschool children progresses rapidly over six months. The slight increase in caries prevalence and dmft in our study may partly reflect this natural disease progression. The observed decrease in dt and increase in ft likely reflect the restorative component of our model, but the absence of a control group prevents us from quantifying the true preventive effect. Third, time effects cannot be excluded. Seasonal changes, public health campaigns, or school activities during the six-month period may have influenced oral health behaviors independently of our model. Furthermore, the proportion of SECC children completing the questionnaire (63.2%) was substantially lower than that of caries-free (90.3%) or ECC (91.6%) children, suggesting that high-risk families may be harder to engage and retain—precisely the population most in need of intervention. In addition, the study was conducted exclusively in Chengdu, an urban setting in western China, and caution is warranted when generalizing these findings to rural areas or regions with differing socioeconomic profiles. An additional methodological consideration concerns the sample size calculation. The *a priori* power calculation was based on a paired t-test for single time-point comparisons, rather than on the GEE models ultimately used. A GEE-specific formula would have been preferable, but its implementation required precise specifications (45) (working correlation structure, within-subject correlation, covariate effects) for which limited prior data existed. Nevertheless, the *post-hoc* assessment confirmed that the achieved sample far exceeded the conservatively adjusted GEE requirement. Therefore, the simplified calculation does not materially affect our interpretation. Future trials should incorporate GEE-specific sample size formulae at the design stage.

A further limitation is the uniform recall schedule (1, 3, and 6 months) applied to all participants, which deviates from CAMBRA's risk-based philosophy requiring intervals tailored to individual risk status (46, 47). The standardized timeline in the study facilitated consistent data collection and observation of intervention effects. Low-risk children typically require 12-month recalls, whereas high-risk children benefit from 3-month intervals to enable early interception of rapidly progressing lesions (48). Thus, the uniform schedule may have attenuated the model's cost-effectiveness and scalability. In routine practice, a risk-stratified approach—annual/biannual recall for low-risk and 3-month recall for high-risk children—would better align with CAMBRA principles. Future implementations should incorporate dynamic risk reassessment at each visit to optimize both clinical appropriateness and resource allocation.

In conclusion, this study successfully established a “family-school-hospital” collaborative oral health management model incorporating personalized guidance, clear care pathways, and behavior-formation tools. The model was associated with increased parental attention to preschool children's oral health and improved parental oral health knowledge. The model also coincided with increases in proactive oral health behaviors in children, and was associated with enhancements in the children's OHRQoL. Further follow-up with a longer duration is warranted to assess the long-term preventive effects of this model. Future studies should incorporate a randomized controlled design to establish causal efficacy, explore strategies to maintain adherence to preventive behaviors beyond six months—particularly for fluoride application and flossing—and evaluate the cost-effectiveness of implementing this collaborative model at scale across diverse populations.

## Data Availability

The original contributions presented in the study are included in the article/Supplementary Material, further inquiries can be directed to the corresponding author.
